# Biophysical principles of pulsed field ablation

**DOI:** 10.1093/europace/euag229

**Published:** 2026-10-21

**Authors:** Francesca Apollonio, Francesca Camera, Micaela Liberti, Caterina Merla, Lluis M Mir, Carmen Pisano, Poompavai Sadasivam, Govindarajan Srimathveeravalli, Maura Casciola, Kars Neven

**Affiliations:** ICEmB at DIET, Sapienza University of Rome, Rome, Italy; National Italian Agency for Energy, New Technologies and Sustainable Economic Development ENEA, Division of Biotechnologies, via Anguillarese 301, Rome 00123, Italy; ICEmB at DIET, Sapienza University of Rome, Rome, Italy; National Italian Agency for Energy, New Technologies and Sustainable Economic Development ENEA, Division of Biotechnologies, via Anguillarese 301, Rome 00123, Italy; Université Paris-Saclay, CNRS, Gustave Roussy, Biologie du cancer et Métabolisme, 94805, Villejuif, France; ICEmB at DIET, Sapienza University of Rome, Rome, Italy; Department of Mechanical and Industrial Engineering, University of Massachusetts Amherst, Amherst, MA, USA; Department of Mechanical and Industrial Engineering, University of Massachusetts Amherst, Amherst, MA, USA; Medical Affairs, Boston Scientific S.p.A., Viale Enrico Forlanini 23, 20134 Milan, Italy; Department of Electrophysiology, Alfried Krupp Hospital, Essen, Germany; Department of Medicine, Witten/Herdecke University, Witten, Germany

**Keywords:** Pulsed field ablation, Electroporation, Irreversible electroporation, Cardiac ablation, Biophysics, Electric field, Lesion formation, Tissue selectivity

## Abstract

Pulsed field ablation (PFA) is emerging as a major advance in cardiac ablation because it induces irreversible electroporation rather than relying primarily on thermal injury. This article provides an overview of the core biophysical principles underlying PFA, focusing on concepts broadly relevant across systems and clinical applications. Externally applied pulsed electric fields interact with biological tissues and affect their transmembrane potential. When a critical threshold is exceeded, nanoscale aqueous pores form in the lipid bilayer, initiating electroporation. Depending on pulse amplitude, duration, number, repetition frequency, waveform, and tissue characteristics, membrane permeabilization may remain reversible or progress to irreversible injury and cell death. This article focuses on the fundamental biophysical principles of electroporation that underpin PFA. The article outlines how membrane charging, cell geometry, and tissue heterogeneity influence electric field distribution and biological response and explains why lesion formation depends on both field intensity and exposure conditions. It also summarizes the molecular basis of pore formation, the potential contribution of lipid peroxidation and membrane proteins, and the downstream processes linking sustained permeability to loss of homeostasis and delayed cell death. Finally, it places cardiac PFA in the broader context of electroporation-based therapies developed in oncology, drug delivery, and gene transfer, while highlighting the remaining need for cardiomyocyte-specific mechanistic evidence to further optimize safety, precision, and durability.

## Graphical Abstract

**Graphical Abstract:**
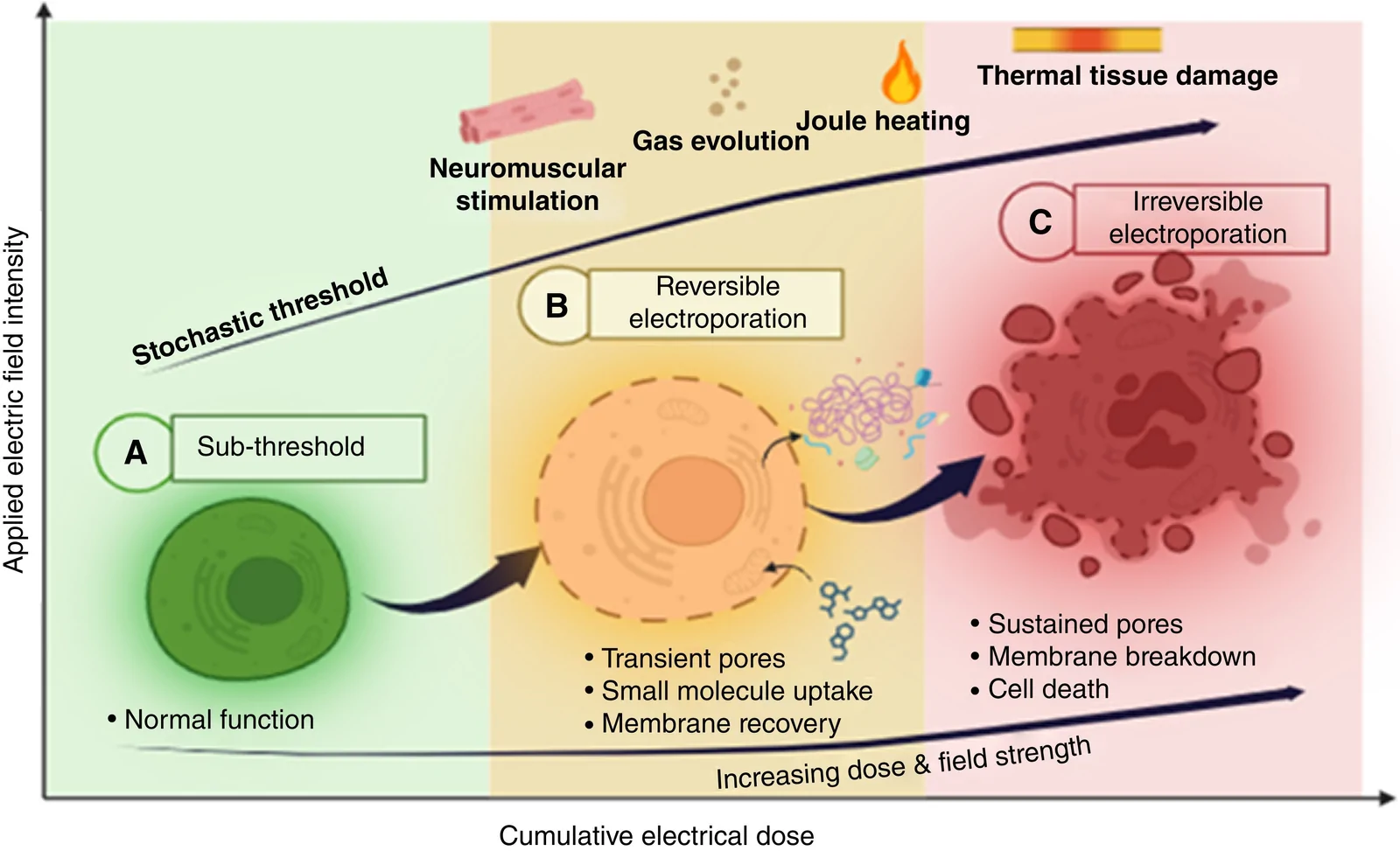

## Introduction

Pulsed field ablation (PFA) has emerged as a transformative energy modality for treating cardiac arrhythmias, introducing a mechanism of action fundamentally different from thermal ablation technologies.^1–6^ Rather than relying on tissue heating or cooling, PFA applies high-intensity, short-duration pulsed electric fields (PEFs) to induce irreversible electroporation (IRE) in myocardial cells. Recent randomized and real-world data have established PFA as a clinically relevant modality beyond mechanistic promise. The ADVENT randomized trial demonstrated non-inferior effectiveness with a favourable safety profile compared with thermal ablation in paroxysmal atrial fibrillation.^7^ Findings supported by large real-world safety analyses report low rates of oesophageal, phrenic nerve, and cerebrovascular injury.^8,9^ Emerging head-to-head comparisons with cryoballoon ablation further suggest that clinical outcomes depend on delivered waveform and dose, reinforcing the need for standardized, evidence-based PFA energy definitions.^10^

The non-thermal basis has important implications for lesion formation, preferential tissue effects, procedural safety, and clinical workflow. A clear understanding of the underlying biophysical principles is therefore essential for interpreting preclinical data, optimizing system design, and translating this technology into safe, effective, and reproducible clinical practice.

This chapter summarizes the mechanistic foundation of electroporation by describing how externally applied PEFs interact with biological tissues, how these interactions lead to electroporation at the cellular level, and how waveform characteristics influence biological outcomes. The concepts introduced here provide the scientific framework needed to understand the PFA engineering choices, tissue effects, and clinical observations discussed in subsequent chapters of this Supplement.

### Scope of this article

This article focuses on the fundamental biophysical principles of electroporation that underpin PFA. Its scope is to present the core biophysical concepts that govern electroporation-based medical applications, with a focus on principles that are universally applicable across PFA systems and clinical indications. Emphasis is placed on electric field–tissue interactions, membrane electroporation mechanisms, and the role of PEF waveform parameters in determining biological response. The discussion draws from the wider electroporation literature developed across biomedical engineering, oncology, and cell biology.

This foundational knowledge serves as the basis for catheter and generator design, lesion characterization, tissue preferential effects, procedural strategies, and clinical outcomes.

### Interaction of electric fields with biological tissues

Biological tissues are electrically heterogeneous structures composed of cells, extracellular matrix, and conductive fluids. When an external electric field is applied, charges redistribute within and around cells, leading to the induction of transmembrane potential (TMP) across the lipid bilayer.^11,12^ The magnitude and spatial distribution of this induced TMP depend on the applied field strength, pulse parameters, tissue conductivity, cell size and orientation, and the local electric field distribution.

At lower electric field strengths, the induced TMP remains below the threshold required to disrupt membrane integrity, and cellular effects are transient and reversible. When the appropriate electric field strength and pulse durations are applied, nanoscale aqueous defects form within the lipid bilayer, a phenomenon known as electroporation.^13–15^ When the extent of membrane disruption exceeds the cell’s ability to recover its functions, IRE occurs, leading to loss of homeostasis and subsequent cell death.^16–18^

This electric field–driven mechanism is central to PFA and distinguishes it from thermal ablation modalities, which require a higher electric field and longer exposure time to occur (*Figure 1*). Because energy deposition is not primarily mediated by resistive heating, tissue effects depend more directly on electric field distribution than on temperature gradients. Understanding how electric fields propagate through complex cardiac anatomy and how cells respond to these fields is therefore essential for predicting lesion formation, safety margins, and tissue response.

**Figure 1:**
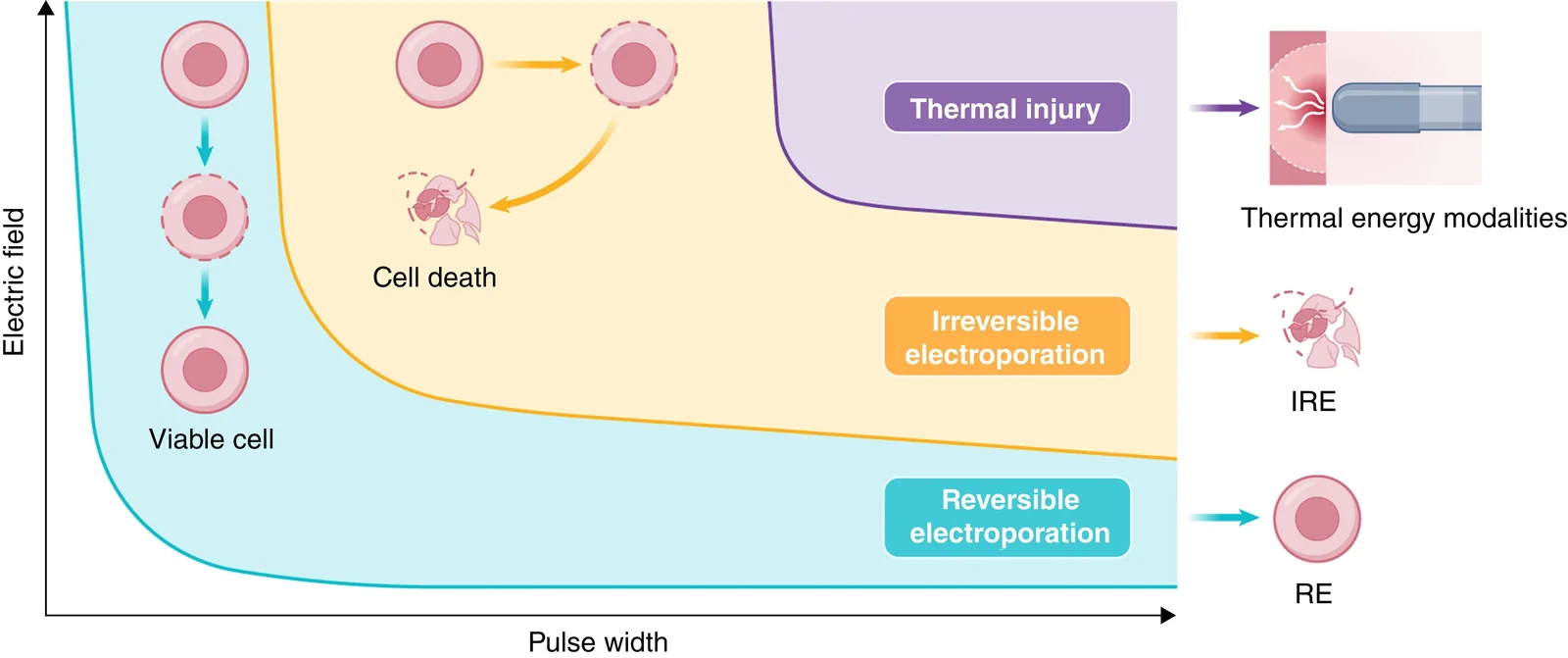
Relationship between electric field strength and pulse width showing transitions from reversible electroporation (RE) to irreversible electroporation (IRE) and, at higher energy delivery, thermal injury.

## Induced transmembrane potential

In PFA, microsecond-range electric fields drive ionic movement across cell membranes, creating TMP that triggers electroporation, whereas much higher-frequency fields act via different mechanisms not used in current cardiac PFA.^19–22^

Charges arising from ionic displacement and, at higher frequencies, dipolar orientation accumulate at the cell membrane, leading to an asymmetric charge distribution across the lipid bilayer, with positive charge on the extracellular side and negative charge on the intracellular side.^22,23^ This charge separation generates an additional voltage across the membrane, superimposed on the physiological resting potential, referred to as the induced TMP. When the externally applied electric signal (i.e. voltage or electric field) is of sufficient amplitude, the induced TMP can destabilize the lipid bilayer, resulting in the formation of transient aqueous defects (electropores) and initiation of *electroporation*.^21,22^ The interaction between externally applied PEFs and biological membranes can be described as a two-step process: electroporation followed by electropermeabilization.^13,22,24^ In brief, electroporation occurs when the induced TMP exceeds a threshold (i.e. electroporation threshold E_th_), triggering a rapid lipid membrane reorganization and the formation of hydrophilic pores during the electric pulse application (see the section ‘Initiation of aqueous pores in lipid bilayers at high transmembrane potentials’ for more details). *Electropermeabilization* then describes the state of increased membrane conductivity and permeability that may persist beyond the electric pulse duration. Depending on the waveform pulse parameters, this state can be transient or permanent.^13,22,25^ The overall phenomenon, comprising electroporation (increase of cell permeability during PEF delivery) and electropermeabilization (increase of cell permeability after PEF delivery), is often referred to as *electropulsation*.^26^

Electroporation thresholds vary among tissues and cell types.^13,22,27,28^ For μsPEF, larger cells tend to accumulate higher induced TMP for a given external field, making them easier to electroporate (see the section ‘Principles of membrane charging, dependence on cell size, geometry, and extracellular electric field distribution’). Nevertheless, recent evidence highlights the role of cytoskeletal structure, membrane proteins, lipid bilayer composition, and the cell-to-nucleus size ratio influencing this threshold, complicating predictions based on size alone.^13,22,29,30^

Independently of the specific electroporation threshold, the development of the induced TMP is limited by how fast the cell membrane can charge, a property that depends on cell and tissue characteristics. This membrane charging time determines how cells respond to different pulse durations and waveforms and is therefore a critical parameter in PFA.^31,32^ Electroporation depends not only on electric field strength but also on how the field is distributed in space and time within the target tissue. Tissue architecture, cell shape, and cell–cell organization strongly influence the locally induced TMP, making its distribution heterogeneous across the tissue. As a result, validated numerical modelling is often required to predict where electroporation occurs in structurally complex tissues.^33,34^

### Principles of membrane charging, dependence on cell size, geometry, and extracellular electric field distribution

From a simplified physical perspective, a cell can be modelled as a spherical dielectric shell, representing the capacitive cell membrane, surrounded by conductive intracellular and extracellular media and exposed to an externally applied electric field.^12,35^ Under this approximation, the membrane behaves as a capacitor, with charge accumulating on its surfaces and the induced TMP increasing over time towards a value determined by cell geometry and tissue conductivity. The induced TMP is not uniform along the membrane but varies spatially, reaching its maximum at the cell poles aligned with the direction of the applied electric field. This spatial distribution has been demonstrated experimentally using voltage-sensitive fluorescent probes.^36^ An established expression for a non-permeabilized spherical cell is:


$$\Delta V_{m}(\theta ,t)=\frac{3}{2}RE_{0}\cos\;\theta (1-e^{-t/\tau ch\arg\;e})$$

Where $\Delta V_{m}$ is the induced TMP, *R* is the cell radius, $E_{0}$ the applied electric field, *θ* the polar angle, and $\tau_{charge}$ the membrane charging time constant. The corresponding passive steady-state TMP is $\Delta V_{m,\mathrm{ss}}(\theta )=\frac{3}{2}RE_{0}\cos\;\theta$. At the cell pole considered in *Figure 2*  (θ=0), $\Delta V_{m,\mathrm{ss}}^{\mathrm{pole}}=\frac{3}{2}RE_{0}$. The membrane charging time constant is defined as:


(1) $$\tau_{charge}=RC_{m}\left(\frac{1}{2G_{ext}}+\frac{1}{G_{cyt}}\right)$$

where $C_{m}$ is the membrane capacitance per unit length, and $G_{ext}$ and $G_{cyt}$ the extracellular and cytoplasmic conductances, respectively.

**Figure 2:**
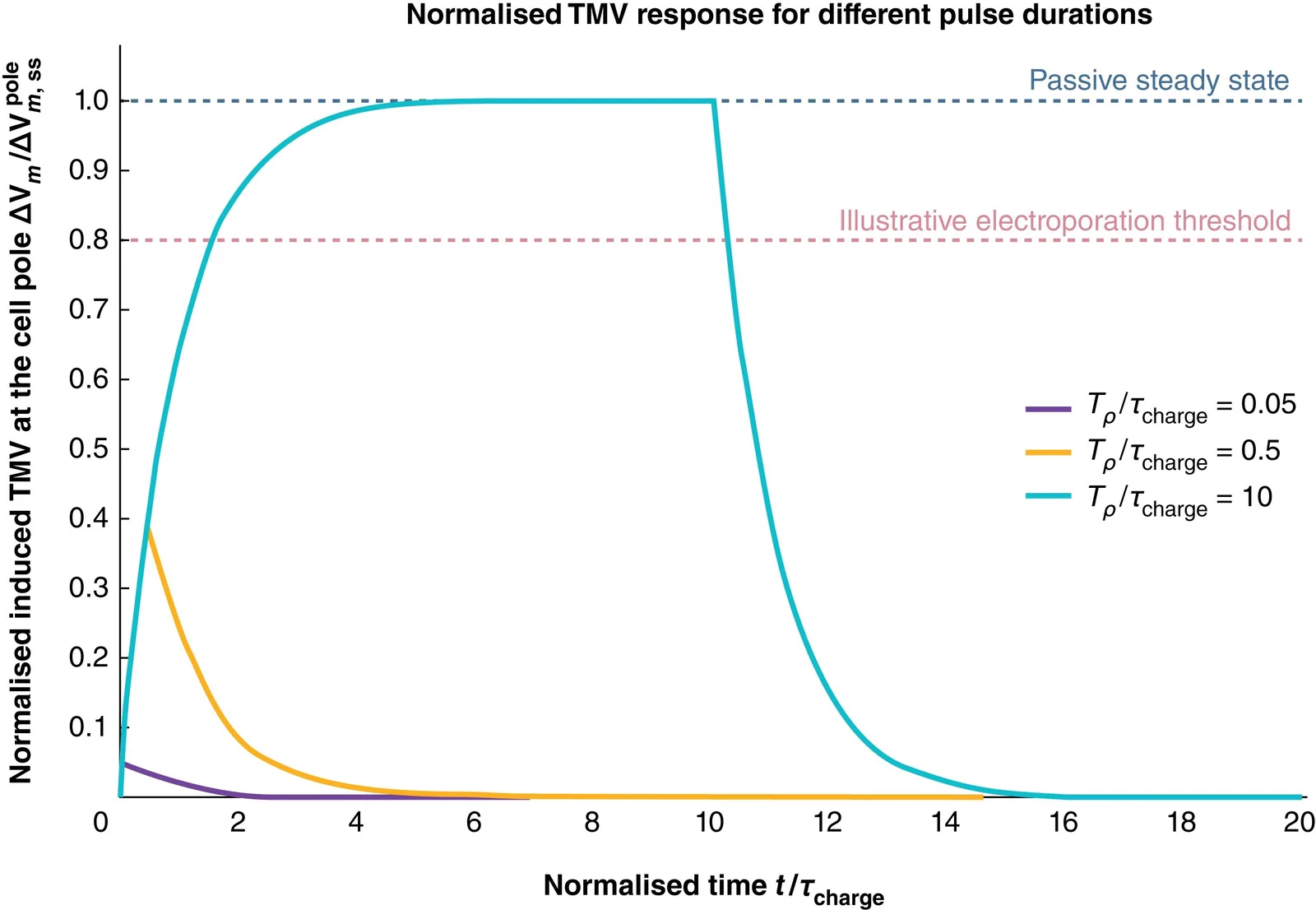
Normalized temporal evolution of the induced transmembrane potential (TMP), $\Delta V_{m}$, at the pole of an intact spherical cell (θ=0) during rectangular electric pulses of different durations. Here, *θ* is the polar angle relative to the direction of the applied electric field, *t* is time, $T_{p}$ is the pulse duration, and $\tau_{\mathrm{charge}}$ is the characteristic membrane charging time constant. Time is normalized to $\tau_{\mathrm{charge}}$, and the induced TMP is normalized to its passive steady-state value at the cell pole, $\left(\Delta V_{\left(m,ss\right)}^{\;pole}=3RE_{0}/2\right)$. The curves illustrate incomplete membrane charging when $\left(T_{P}\leq \tau_{ch\arg\;e}\right)$ and quasi-steady-state charging when $\left(T_{p}\gg \tau_{ch\arg\;e}\right)$. The dashed blue line indicates the normalized passive steady-state TMP, whereas the dashed red line denotes an illustrative normalized electroporation threshold and should not be interpreted as a universal or cell-specific threshold.

Thus, larger cells exhibit longer $\tau_{charge}$ and higher steady-state induced TMP, and they require longer electric pulses to reach maximal TMP.^32–34^

When electroporation occurs, membrane conductance increases due to the formation of aqueous pores in the lipid bilayer, which allows enhanced ionic transport across the membrane, which shortens the charging time and alters TMP spatial distribution:


(2) $$\tau_{charge}=RC_{m}\frac{\left(G_{cyt}+G_{ext}\right)}{2G_{cyt}G_{ext}+RG_{m}\frac{\left(G_{cyt}+2G_{ext}\right)}{d}}$$

where $G_{m}$ is the membrane conductance and *d* the membrane thickness.^32^ Physiological membrane charging times are typically in the microsecond range, indicating that PEF of a few microseconds can readily induce TMP values exceeding the critical electroporation threshold (commonly < 1 V, though broader ranges up to ≈1 V are reported depending on conditions).^37,38^ Experimental studies confirm these predictions, showing that membrane permeabilization depends on electric field strength and pulse duration, as well as on cell geometry and orientation relative to the applied field.^39^

For non-spherical geometries, such as the elongated morphology of cardiomyocytes, the description given by Eq. (1) becomes substantially more complex. In such cases, the cell can be approximated as an ellipsoid, and the corresponding analytical formulation of the TMP has been derived and discussed in detail elsewhere.^40–42^ Although the spherical approximation neglects cell elongation and other geometrical features, it remains a valuable and extremely simple tool for obtaining first-order estimates of the TMP. Indeed, deviations between spherical and ellipsoidal models for representative cellular aspect ratios over a frequency range extending up to a few MHz remain below one order of magnitude^43^ depending on cell aspect ratio. Therefore, while more sophisticated geometrical descriptions are required for accurate quantitative predictions, the spherical model can still provide reasonable estimates of TMP under many practical conditions. However, a rigorous assessment of the TMP requires mandatory numerical modelling, typically based on finite-element methods, to account for realistic cell morphologies and cell–cell aggregation, and to accurately predict the resulting distributions of TMP and membrane poration, as demonstrated in the studies by De Angelis et al. and Golberg et al.^33,34^

In these cellular models, the dielectric permittivity and electrical conductivity of the various cell compartments are typically derived from literature data available for different cell types.^35,44–48^ However, the anisotropy of cardiac tissue, which arises from the elongated morphology and preferential alignment of cardiomyocytes, should be accounted for when performing dosimetric assessments at higher levels of biological organization, such as tissue and organ models. This anisotropy strongly affects the distribution of the induced electric field and therefore plays a key role in determining optimal electrode configurations as well as the size and geometry of the resulting PFA lesions.

### Influence of pulse duration and tissue heterogeneity on the magnitude and spatial distribution of induced voltage

PEF duration controls the extent of charge redistribution across dielectric interfaces, particularly the plasma membrane.^36,39^

For electric pulse durations longer than the membrane charging time, induced TMP approaches a quasi-steady-state value governed by geometry and conductivity contrasts. When applied electric pulses are comparable to or shorter than the membrane charging time, membrane charging is incomplete, peak induced TMP is reduced, and displacement currents dominate (*Figure 2*).^49–51^ In this regime, dielectric properties of cell/tissues become critical, and electric fields may also penetrate the cell cytoplasm.^35^

Tissue heterogeneity further complicates voltage distribution through Maxwell–Wagner polarization. This phenomenon arises from the accumulation of charges at interfaces between regions with different electrical conductivity and permittivity. This interfacial polarization introduces a frequency-dependent redistribution of the electric field, thereby affecting local TMP and current density.^42^ Interfaces between regions of different conductivity and permittivity (e.g. vessels, fibrous septa, and fat/muscle boundaries) can locally amplify or attenuate the local electric field, generating ‘hot spots’ or ‘sinks’. Low-conductivity and permittivity regions tend to concentrate electric fields (such as the pericardium), whereas highly conductive regions shunt current, decreasing the induced electric field (such as blood). These effects are three-dimensional and tissue orientation-dependent, often requiring numerical solutions of Maxwell’s equations again to get the temporal and spatial distribution of the induced electric field and voltage.^33,34,39^

## Electropulsation as the core phenomenon behind PFA: physico-chemical basis of pore formation

To safely and effectively use PFA, it is essential to understand how electric fields interact with cardiac tissue at the cellular level. The physical basis of electroporation is rooted in molecular-scale membrane processes, while the distinction between reversible and irreversible outcomes emerges progressively at higher organizational levels (*Figure 3*). Molecular events define the probability and stability of pore formation, but cellular recovery, tissue architecture, and electric field distribution determine the macroscopic and clinical effects observed in PFA. This multilevel interpretation is consistent with recent clinical–mechanistic analyses of PFA, which emphasize how electroporation thresholds, tissue-specific properties, and cumulative pulse delivery jointly determine lesion formation and safety profiles at the organ level.^52^

**Figure 3:**
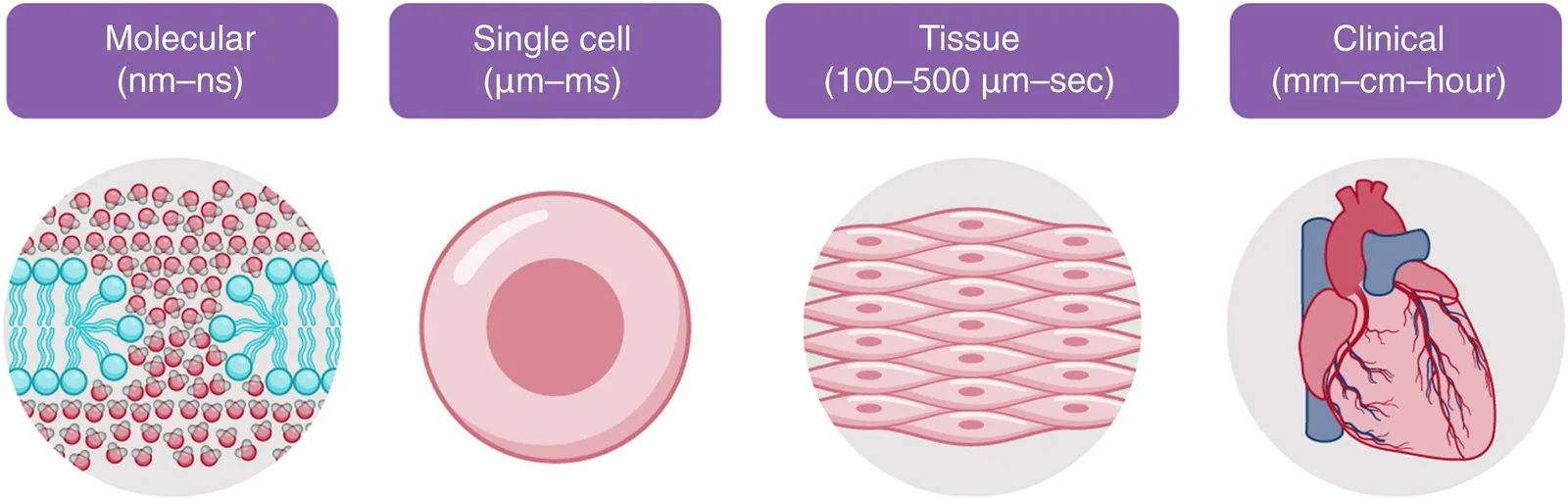
Multiscale framework of electroporation in pulsed field ablation. Schematic representation of the hierarchical organization of electroporation phenomena across spatial scales. At the molecular level, electric fields stabilize transient aqueous pores in lipid bilayers in a stochastic manner. At higher levels of organization, cellular recovery mechanisms, tissue architecture, and electric field distribution modulate the transition from reversible to irreversible electroporation. Clinical lesion formation in pulsed field ablation emerges from the integration of these multiscale processes rather than from a single deterministic membrane threshold.

### Initiation of aqueous pores in lipid bilayers at high transmembrane potentials

Intense electric fields induce high TMPs that destabilize the lipid bilayer, forming aqueous pores.^17,22,53,54^ Classical continuum theories describe this as an electric-field–dependent lowering of the free-energy barrier, explaining threshold behaviour and macroscopic permeability changes.^20,51,55,56^ However, direct electrophysiological measurements on planar lipid bilayers have challenged the quadratic field-dependence predicted by this classical model, showing instead that the activation barrier for pore formation decreases with the absolute value of the electric field rather than its square, a result more consistent with a field-driven reorganization of the lipid–water interface than with the standard electromechanical picture.^57^ Molecular dynamics (MD) simulations provide nanoscale insight, showing that water dipoles first align at the lipid–water interface, forming ‘aqueous fingers’ that penetrate the hydrophobic core and guide lipid headgroup reorientation to stabilize the pore (*Figure 4*).^14,58–61^ These events occur without membrane proteins, indicating electroporation is an intrinsic lipid property.^61,62^ Although MD studies are limited in timescale and complexity, their findings align with continuum models and experimental evidence within a multiscale framework.^22,63^

**Figure 4:**
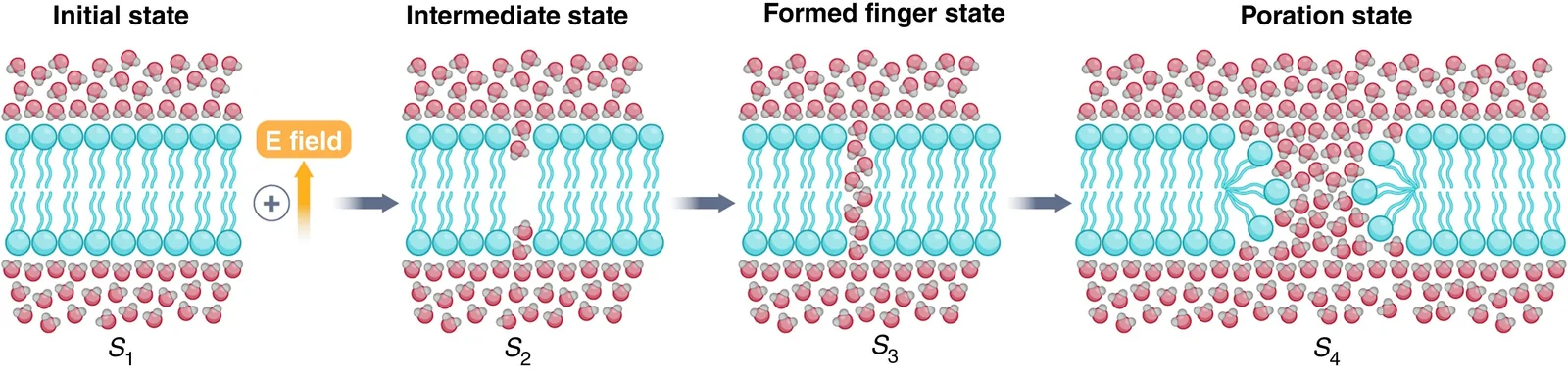
Sequence of molecular events leading from aqueous defect initiation to pore formation in lipid bilayers under high TMPs. The schematic illustrates the progression from an intact membrane (S1), through intermediate states characterized by field-driven water penetration and aqueous finger formation (S2 and S3), to the formation of a continuous hydrophilic pore across the membrane (S4). Adapted from Marracino et al.^21^.

### Lipid peroxidation and its impact on cell membrane

The molecular composition of the lipid bilayer and chemical modifications, such as lipid peroxidation, can significantly alter membrane structure, hydration, and mechanical properties, creating a state more susceptible to electroporation without directly forming pores.^25,64,65^ This view has, however, been challenged by molecular dynamics simulations showing that sufficiently high degrees of lipid oxidation can nucleate aqueous pores directly, even in the absence of an applied electric field.^66^ Conversely, quantitative free-energy calculations indicate that the increase in ionic permeability and conductance conferred by peroxidized lipids alone, although substantial (up to several orders of magnitude), is insufficient to account for the full extent of membrane permeabilization measured experimentally in electropermeabilized cells,^67^ suggesting that lipid oxidation likely acts synergistically with, rather than as a substitute for, field-driven pore formation. Oxidative reactions introduce polar groups into the hydrophobic core, disrupt lipid packing, increase polarity, and partially hydrate the membrane, reducing mechanical stability and lowering the energetic barrier for pore formation^65,68^ (*Figure 5A*). PEFs can indirectly enhance these effects by increasing intracellular reactive oxygen species, promoting lipid peroxidation over hours^69,70^ (*Figure 5B*). These delayed oxidative changes are mechanistically distinct from the immediate, field-driven pore formation but can influence membrane properties over longer timescales.^64,71^

**Figure 5:**
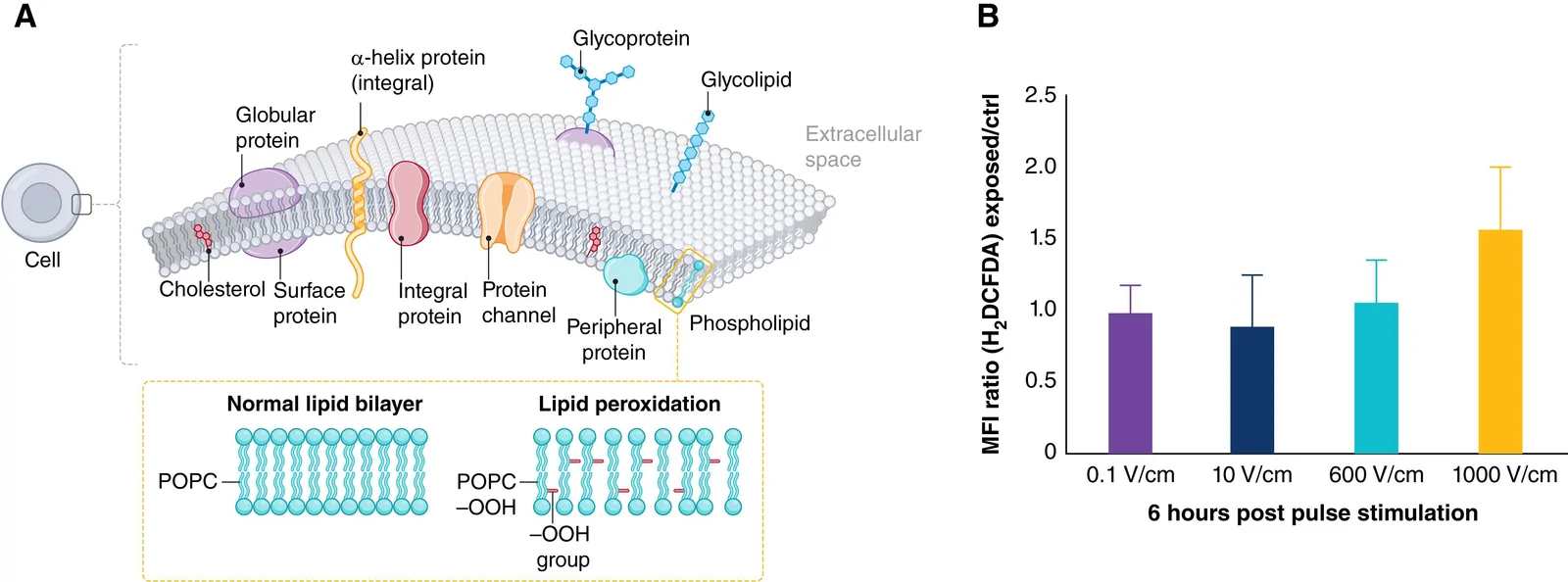
Modulatory role of lipid peroxidation in membrane susceptibility to electroporation. (*A*) Schematic representation of membrane lipid peroxidation. Oxidative modification of unsaturated phospholipid acyl chains introduces polar hydroperoxide groups (−OOH), locally disrupting bilayer packing, increasing membrane polarity, and facilitating water penetration into the lipid core. These changes alter membrane material properties without directly inducing pore formation (Páez-Pérez et al.^27^). (*B*) Experimental evidence of increased intracellular oxidative stress following pulsed electric field exposure, shown as the mean fluorescence intensity (MFI) ratio of the ROS-sensitive probe H_2_DCFDA measured 6 h after stimulation at increasing electric field strengths adapted from.^70^

### Potential involvement of membrane proteins

While the lipid bilayer is the primary substrate for electropore formation, membrane proteins can locally modulate or bias electroporation.^22,61,62^ Experimental studies and molecular dynamics simulations show that electric fields induce structural changes in proteins and reorganize protein-associated water, creating pathways that influence pore nucleation.^26,72–74^ Cellular membranes, with their dense transmembrane proteins *Figure 5A*, introduce local dielectric and structural heterogeneities that affect electroporation dynamics without being required for pore formation.^22,75^ MD simulations consistently highlight the role of water polarization as a unifying mechanism (*Figure 6*): above threshold electric fields, water dipoles align and form localized aqueous perturbations near proteins, often preceding lipid bilayer poration.^61,74,76^ In cardiac cells, abundant ion channels and transporters may therefore contribute to spatial heterogeneity in electroporation susceptibility.^22^

**Figure 6:**
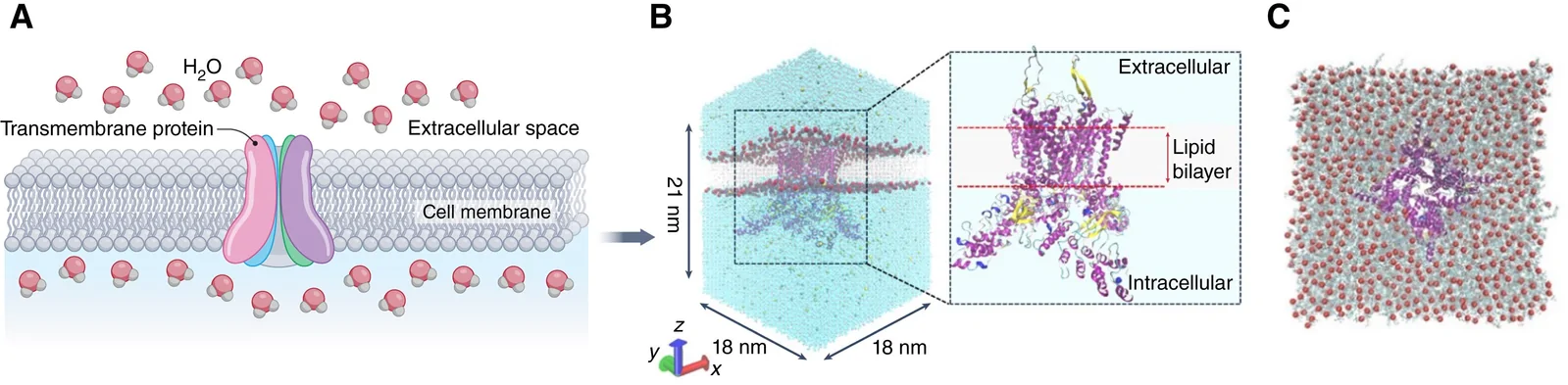
Conceptual and molecular dynamics representation of protein-associated modulation of electroporation. (*A*) Conceptual schematic linking a simplified representation of a transmembrane protein embedded in a lipid bilayer with its atomistic molecular dynamics model. (*B*) Atomistic view showing the corresponding simulation box used to investigate protein–membrane assemblies under electric field exposure with a magnified side view of the transmembrane protein spanning the lipid bilayer. (*C*) Top-down (xy-plane) view of the same protein–membrane assembly in its pre-pore, intact state, showing no aqueous pore formation, provided as a reference baseline for comparison with the field-induced pore formation sequence illustrated in *Figure 4*.

### Electric field strength and exposure time drive variability in response

Electroporation is a probabilistic process rather than a deterministic event. The formation and stabilization of nanoscale aqueous pores depend on lipid rearrangement, lipid oxidation, and protein–membrane interactions, with pore likelihood, size, and lifetime increasing nonlinearly with electric field intensity and exposure time.^22^ MD simulations show substantial variability in the timing, location, and size of pores, even under identical TMPs, reflecting the stochastic stabilization of thermally fluctuating water and lipid molecules.^14,71,77^

Higher electric field strength lowers the energetic barrier for pore formation, increasing the probability of larger and longer-lived pores, while longer exposures cumulatively raise the chance that transient pores evolve into metastable or irreversible pathways.^51,78^ At the tissue level, this behaviour gives rise to an apparent permeabilization threshold emerging from the collective response of many cells, rather than from a sharply defined molecular transition.^62,79^

In PFA, this probabilistic framework explains why the extent of reversible electroporation vs. IRE depends on both pulse amplitude and duration across the targeted tissue volume, forming the basis for dose–response relationships and lesion formation discussed in the section ‘Lipid peroxidation and its impact on cell membrane’.

## Reversible electroporation

Reversible electroporation occurs when the electric field transiently increases membrane permeability, but the cell’s repair mechanisms restore membrane integrity and homeostasis afterwards. Under these conditions, the membrane reseals within minutes to hours, and the cell can recover its normal functions, making this regime useful for molecular delivery and gene transfection applications.^80^ Survival after reversible electroporation depends on pulse parameters such as electric field strength, pulse duration, waveform, and number of pulses, as well as intrinsic cell characteristics including size, shape, and cytoplasmic conductivity.^81–83^ Cells that experience only limited permeabilization tend to maintain viability if electric stress does not overwhelm their repair capacity.

### What means ‘reversible electroporation’

Historically, before ‘electroporation,’ the delivery of short and intense PEFs to microorganisms and vegetal products was known as ‘dielectric breakdown,’ illustrating by this term the capability of the electric pulses to destroy almost any tissue and cell. When E. Neumann published in 1982^15^ the seminal article in which he coined the term ‘electroporation’, the demonstration was made that, after the exposure of cells to DNA and to short and intense electric pulses, some of the cells could survive and incorporate external molecules such as the DNA previously added in the electropulsing medium.^80^ later found electrical parameters allowing for the reversible permeabilization of all the *in vitro* treated cells with minimal cell loss (cell death). The same group found that reversible electropermeabilization was an efficient physical method for the vectorization of drugs and nucleic acids.^84,85^

Reversible electroporation is defined operationally by post-pulse recovery and survival under given conditions. By itself, it does not ‘kill’ the cells, and the only ‘visible’ effect is a transient (and limited) swelling of the treated cells. To detect the reversible permeabilization of the membrane, a non-permeant probe (a molecule that does not cross the intact plasma membrane and that is not transported actively across it) must be added outside the cells before the delivery of the electric pulses. Depending on its physico-chemical characteristics, and on the strength of the signal generated by the probe when it reaches the cell inside, some probes can also be added just after the delivery of the electric pulses since the permeabilized state lasts for up to a few minutes (depending on the electric pulse parameters).

### Characteristics of the reversible regime

#### Conditions allowing cell survival despite transient loss of membrane integrity

The two main conditions to respect for achieving reversible electroporation of the membranes are an appropriate working medium and an appropriate set of electrical parameters.

Indeed, the external medium must be isoosmotic with the cell’s internal medium. Otherwise, after the electric pulse’s delivery, the movement of ions or solutes across the membrane, and the subsequent movement of water, will cause the swelling or shrinking of the cells, which, if excessive, will necrotize the cells. *In vitro*, this condition must absolutely be respected, while *in vivo*, this is not an issue as the body fluids (blood, serum, etc.) are all equilibrated in terms of ion composition. It must be noted that, although ion concentrations are important, the critical consideration in this context is osmolarity (the concentration of solutes), rather than conductivity (which depends on the concentration of ions). *In vitro*, it is possible to play with osmolarity and conductivity independently, and both parameters must be under control.

Four PEF waveform parameters are important: the pulse amplitude, the duration of each pulse, the number of pulses, as well as the frequency of repetition of the pulses. All the parameters must be operated within well-defined ranges of values, which also depend on the characteristics of the target cells (size, shape, orientation with respect to the electric field direction, etc.).

#### Temporary nature of membrane permeabilization

During electroporation, cells undergo chemical changes over different time scales. Some occur during pulse delivery and in the minutes immediately afterwards, directly affecting the membrane, whereas others are associated with increased membrane permeability and membrane repair, processes that may continue for minutes to hours. Studies by Lluis M. Mir’s group using mass spectroscopy, Coherent Anti-Stokes Raman Spectroscopy, and Terahertz spectroscopy showed that reversible electroporation causes primary lipid oxidation, increased membrane water content lasting minutes, and leakage of small molecules.^25,28,86^ Principal component analysis or latent variable analysis of the Raman spectra revealed that reversible electroporation restores membrane properties, while IRE produces lasting changes, indicating it involves qualitatively different structural defects rather than simply an accumulation of reversible pores.^26,86^

#### Irreversible outcomes despite membrane resealing

Cells may still undergo death even when the plasma membrane reseals after electroporation. This can happen when poration triggers downstream pathways such as calcium overload, mitochondrial dysfunction, reactive oxygen species generation, or disruption of critical cellular processes.^87^ Electroporation-induced loss of intracellular molecules, impaired homeostasis, or secondary biochemical damage contribute to lethality, indicating that irreversible outcomes are not solely due to failure of membrane resealing but can arise from effects initiated during reversible poration that overwhelm cellular survival mechanisms.

## Irreversible electroporation

IRE denotes a regime of PEF exposure in which electrically induced injury exceeds the cellular capacity for recovery, resulting in loss of viability.^22^ IRE is commonly characterized by persistent membrane defects and failure of membrane resealing following pulse delivery. However, restoration of membrane integrity does not necessarily imply long-term cell survival, as cells may still undergo delayed death through secondary processes including calcium overload, mitochondrial dysfunction, metabolic exhaustion, oxidative stress, and disruption of ionic homeostasis. Cellular fate following electroporation therefore exists along a continuum and depends not only on membrane recovery but also on the ability of the cell to re-establish intracellular homeostasis after exposure. In IRE, sustained permeability, progressive metabolic stress, and failure of these recovery mechanisms ultimately drive irreversible injury and cell death (*Figure 7*).

**Figure 7:**
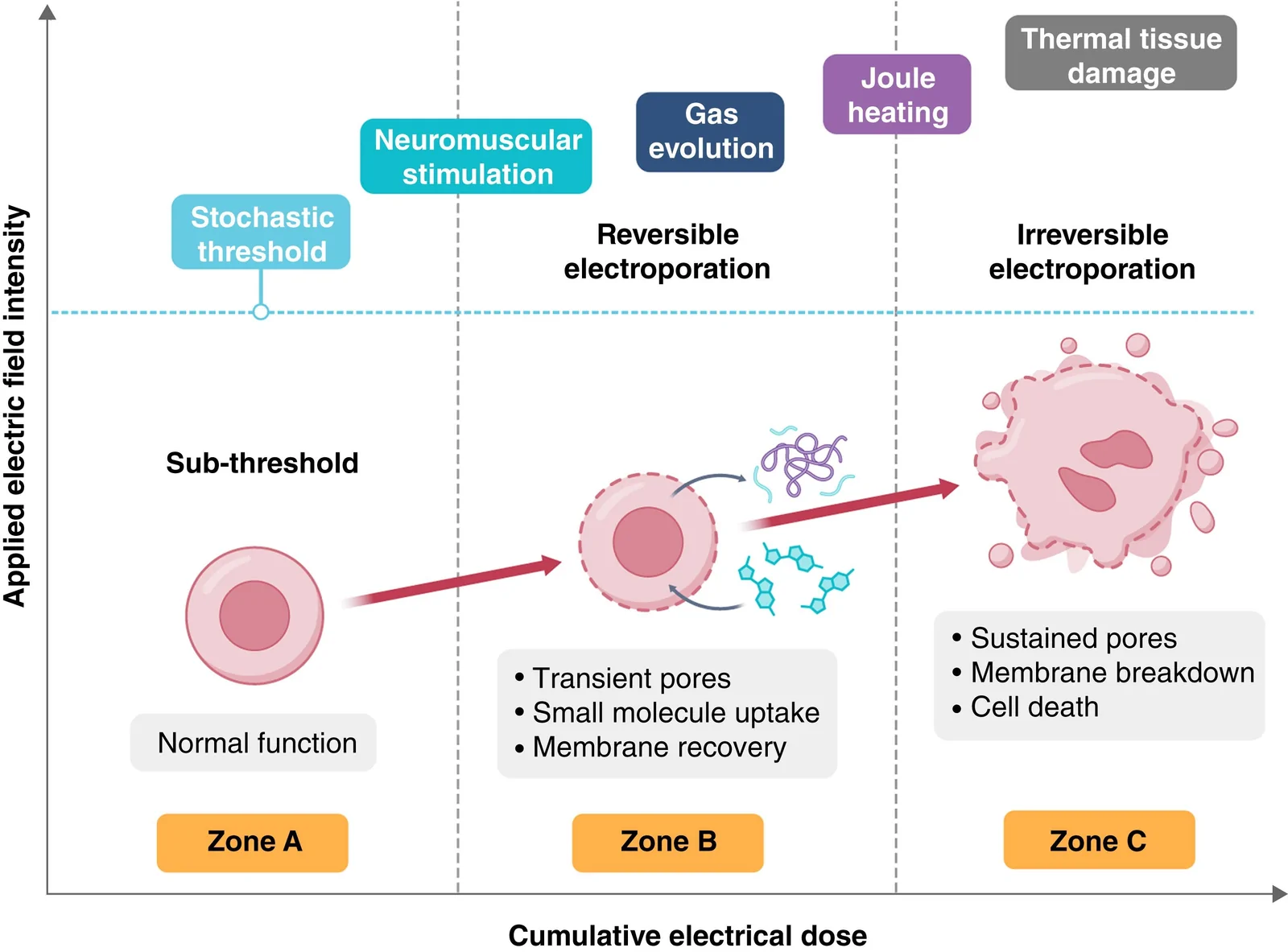
Transition from reversible to irreversible electroporation as a function of applied electric field intensity and cumulative electrical dose. Cellular responses progress from sub-threshold conditions (Zone A), through reversible electroporation with transient membrane permeabilization and recovery (Zone B), to irreversible electroporation associated with loss of cellular recovery capacity and progression towards cell death (Zone C). The boundary between reversible and irreversible effects is stochastic, reflecting biological variability. Higher doses may induce side effects such as Joule heating, gas evolution, and neuromuscular stimulation.^88–90^ Protocols in PFA are intentionally designed to exceed cellular recovery capacity to induce controlled, non-thermal tissue ablation. This energy modality enables spatially confined lesion formation while preserving extracellular matrix architecture and minimizing collateral damage to adjacent structures.^90^

While both cancer therapy and cardiac PFA utilize electroporation-based tissue selectivity, they do so through different mechanisms. In cancer treatments, some studies suggest differences in the susceptibility of malignant and non-malignant cells under certain electroporation conditions: the electric field parameters are set to destroy all cellular components (malignant and healthy) within a targeted volume while sparing the non-cellular collagen scaffolds of blood vessels, nerves, and ducts.^18,91–95^ Conversely, tissue selectivity in cardiac PFA relies on cellular susceptibility thresholds. Cardiomyocytes exhibit different electroporation susceptibility thresholds than adjacent structures.^90^ This allows clinicians to target myocardial tissue while minimizing the risk of collateral damage.

### Transition from reversible to irreversible states

The shift from reversible to IRE is progressive, reflecting the declining ability of cells to repair electrically induced membrane damage rather than a sharply defined boundary. In reversible electroporation, PEFs induce transient membrane permeabilization that allows molecular transport while preserving the cells’ capacity to reseal, maintain ionic gradients, and survive. As cumulative exposure increases, recovery efficiency steadily declines.^22^

Higher electric field amplitudes, longer pulse durations, more pulses, or prolonged exposure convert transient permeabilization into persistent defects. This transition causes sustained ionic flux, osmotic imbalance, and collapse of electrochemical gradients.^96^ Waveform characteristics, including monophasic, biphasic, or high-frequency bipolar pulses, modulate this trajectory by affecting membrane charging and cumulative stress, but irreversible outcomes consistently occur when repair capacity is exceeded.^97^

Biological and tissue factors such as cell size, membrane composition, resting potential, and tissue heterogeneity further shape the transition between reversible electroporation and IRE. This variability produces spatially heterogeneous responses resulting in irreversible injury in high-dose regions and reversible effects nearby.^98^ This spatial gradient is highly relevant to the clinical efficacy of PFA. Peripheral zones subjected to sub-lethal, reversible electroporation are transiently stunned immediately post-procedure, showing an acute loss of excitability and mimicking a successful ablation. However, because these cells recover their membrane integrity and electrical conduction over hours to days, this peripheral zone can fail to achieve permanent block, creating an incomplete lesion that acts as a proarrhythmic substrate.^99^ PFA technologies must therefore be optimized to maximize the irreversible ablation volume while minimizing this surrounding zone of reversible, sub-lethally affected tissue.

#### Thresholds beyond which membrane repair fails due to excessive electric stress

IRE occurs when membrane repair mechanisms fail after PEF exposure, marking the shift from transient permeabilization to persistent defects and loss of homeostasis. Cells may still die even if membranes reseal if permeability overwhelms recovery capacity. (Arena et al., 2011) This repair failure involves excessive pore density, pore coalescence, irreversible lipid rearrangement, cytoskeletal decoupling, disrupted membrane–protein interactions, impaired endocytic repair, and local membrane phase changes. Tissue-level modelling and experimental mapping reveal irreversible injury in regions exceeding the threshold and sub-lethal electroporation effects in adjacent peripheral tissues.^91,93,100^

The threshold for cell death is governed by the combined, non-linear interaction of different parameters including electric field strength, pulse duration, number of pulses, repetition frequency, and waveform types.^81^ Rather than depending on a single parameter the threshold is determined by the cumulative electrical dose and total energy delivered.^101^ Recent studies demonstrate that this threshold is highly dependent on tissue-specific susceptibilities and external environment properties. Myocardium is particularly sensitive to electroporation damage compared to adjacent tissues,^102^ showing a graded response to voltage and pulse number changes.^103^ Furthermore, constant power PEFs have been shown to predict electroporation area and treatment outcomes in a conductivity-dependent manner, overcoming patient-to-patient variability.^104^ Precise control of pulse conditions is essential to achieve predictable and safe clinical outcomes.^105–107^

### Cellular responses following irreversible electroporation

IRE initiates a cascade of cellular responses rather than constituting an instantaneous destructive event (*Figure 8*). Cellular outcomes depend on the overall severity of the electrical insult, which is determined collectively by pulse amplitude, duration, waveform, pulse number, repetition frequency, and tissue-specific factors and on the cumulative electrical dose delivered, rather than on any individual parameter.^108^ During IRE, cells first experience primary physical consequences of sustained membrane permeability, which subsequently propagate into secondary biological processes that evolve over timescales ranging from minutes to hours. Tissue-specific factors, including extracellular matrix composition, cell density, and perfusion, modulate the kinetics of ionic redistribution, metabolic stress, and lesion maturation.^109^

**Figure 8:**
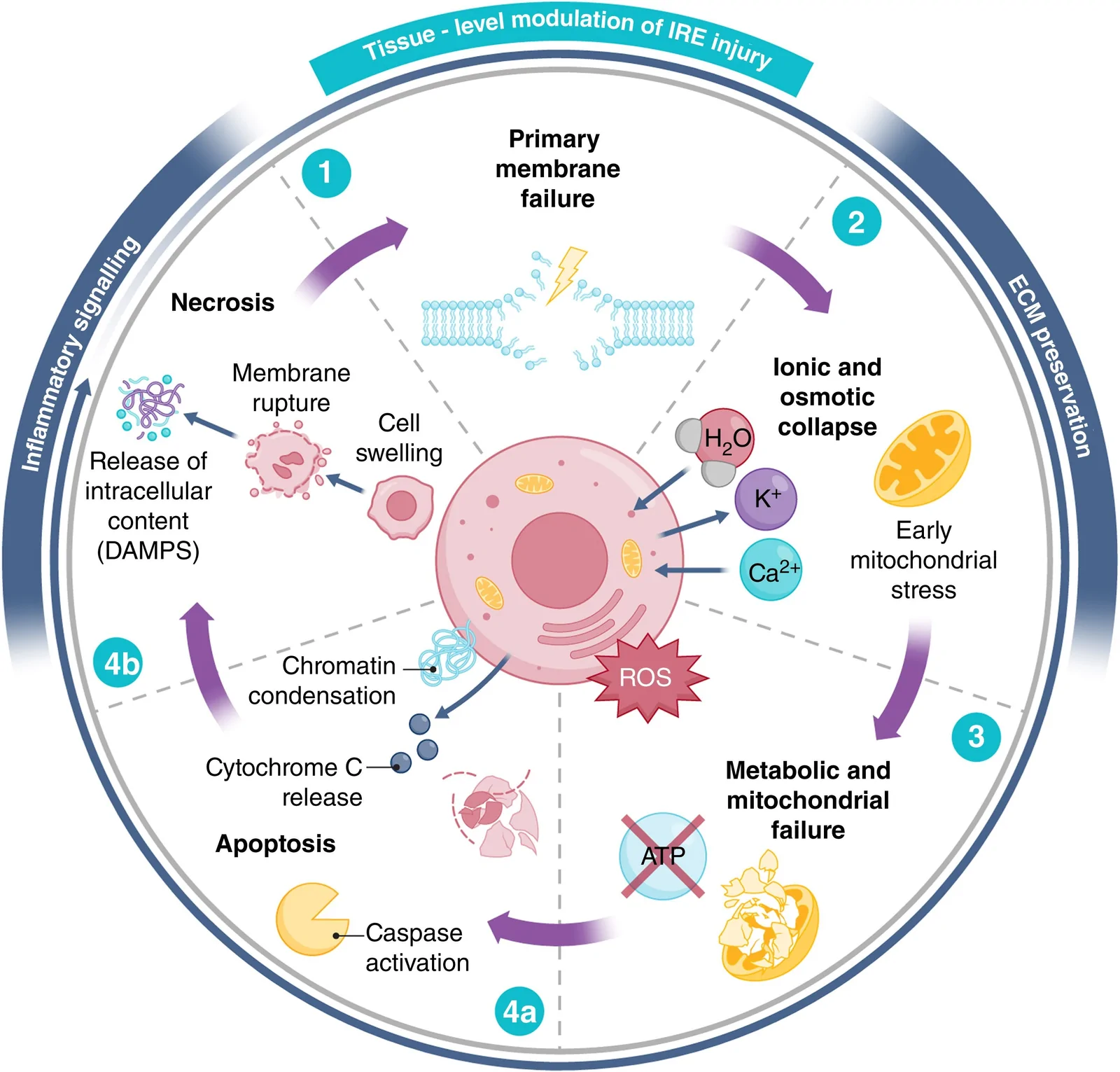
Cellular responses after irreversible electroporation. Following application of a pulsed electric field, a cell undergoes sustained membrane permeabilization, initiating a cyclic injury cascade. This includes (1) primary membrane failure, (2) ionic and osmotic collapse driven by ion flux and water influx, (3) metabolic and mitochondrial dysfunction with ATP depletion and oxidative stress, and (4) energy-dependent cell death, progressing to apoptosis or necrosis depending on injury severity. Tissue-level factors modulate the extent and evolution of injury during PFA.

#### Primary loss of homeostasis from sustained permeability and osmotic imbalance

Sustained membrane permeabilization immediately disrupts ionic gradients and water homeostasis. Uncontrolled fluxes of sodium, potassium, calcium, and water across the plasma membrane result in osmotic swelling, blebbing, and mechanical stress on cellular structures. The resting TMP collapses rapidly, impairing the function of energy-dependent ion pumps such as Na^+^/K^+^-ATPase and Ca^2+^ transporters that are required to maintain electrochemical gradients. These early events, occurring within seconds to minutes following pulse delivery, represent the initiating phase of irreversible cellular injury and establish the conditions for subsequent metabolic and structural failure.^22,98^

#### Secondary downstream processes including metabolic depletion and delayed cell death

Following the initial loss of homeostatic control, cells experience progressive metabolic depletion as ion pumps attempt to restore disrupted gradients in the presence of persistent membrane permeability.^13,22,110^ Sustained ATP consumption, intracellular calcium accumulation, and mitochondrial dysfunction amplify cellular stress and compromise energy production.^111–113^ Depending on the magnitude and duration of metabolic collapse, cells may engage regulated cell death pathways, including apoptosis, necroptosis, or pyroptosis, or alternatively, when the energy depletion is severe, cells progress towards necrotic outcomes characterized by membrane rupture and release of intracellular contents.^114–116^

Delayed cellular responses develop over hours to days and contribute to lesion maturation at the tissue level.^92,93,117^ Persistent metabolic stress can promote secondary processes such as reactive oxygen species generation, inflammatory signalling, and immune cell recruitment, thereby shaping the local tissue environment.^118–120^ As a result, IRE-induced lesions frequently exhibit a combination of apoptotic, necrotic and regulated lytic features, reflecting the interplay between immediate electroporation-induced injury and downstream biological responses.^16,115,119^ Spatially, these effects mirror gradients in cumulative electrical dose, with irreversible injury localized to the lesion core and sub-lethal, potentially reversible effects observed at the periphery^18,91,93^; As discussed already, delayed recovery within these peripheral, reversibly electroporated regions may contribute to incomplete lesion maturation and an increased risk of arrhythmia recurrence.

### Safety considerations and side effects of IRE-based tissue ablation

Even though IRE-based ablation therapies are designed to ablate tissue nonthermally, improper waveform settings, suboptimal catheter geometry, or uneven electric field distribution can lead to unintended side effects.^109,121,122^ These include localized temperature rises (thermal hotspots) resulting from Joule heating under high cumulative energy doses, and electrochemical reactions at the electrode–solution interface that generate microbubbles.^123,124^ In addition, high-voltage pulses can cause neuromuscular stimulation and severe muscle contractions if the electric field extends into areas of reversible electroporation where peripheral motor nerves are polarized.

To mitigate these side effects, contemporary cardiac PFA utilizes biphasic and high-frequency (H-FIRE) pulse trains instead of long monophasic pulses. Biphasic waveforms alternate polarization rapidly, preventing sustained nerve excitation and significantly reducing muscle contraction and pain.^125^ Rapid charge cancellation and optimized pulse-repetition frequencies minimize gas evolution and bubble formation while maintaining therapeutic myocardial ablation volumes.^123,124^ Careful optimization of pulse parameters, catheter design, and procedural workflow remains essential to keep these off-target effects to minimal volumes.^123,124^ Detailed strategies for mitigation in PFA applications are discussed in other sections of this supplement.

## Historical applications outside the heart

This section summarizes established non-cardiac applications of electroporation, including electrochemotherapy, IRE for tumour ablation in the liver, pancreas, and prostate, and gene-delivery approaches in skeletal muscle. These applications represent important steps in the broader evolution of electroporation-based therapies and form the scientific and clinical foundation for cardiac PFA. Fundamental discoveries in reversible electroporation, electrochemotherapy, gene electrotransfer, and later IRE established the biological principles and clinical feasibility of using PEFs to modulate or destroy living tissues in a controlled manner. Decades of experience across these fields have also provided valuable insights into electroporation mechanisms, dose–response relationships, dosimetry, safety, tissue-selective effects, and strategies to minimize off-target effects.

### Paving the way to clinical applications of the delivery of short and intense electric pulses to PFA

#### Preclinical precedents

As described in the section ‘Reversible electroporation’ of this article, the group led by Mir systematically optimized electric pulse parameters *in vitro* to achieve transient cell membrane permeabilization with minimal cell loss.^80^ They further demonstrated that reversible permeabilization increased the cytotoxicity of the anticancer drug bleomycin by several orders of magnitude.^85^

Based on the low intrinsic toxicity of the electric pulses themselves, these findings were translated *in vivo* by administering bleomycin intravenously and applying locally optimized electric pulses to tumours in mouse models. In 1991, this approach led to the first successful treatments of mice bearing superficial tumours using the combination of bleomycin and reversible electroporation, with complete tumour regression achieved after a single treatment in the initial experiments. Importantly, these studies also demonstrated the high safety of the procedure, which the authors termed electrochemotherapy (ECT).^126,127^

#### Electrochemotherapy of tumours in other organs such as liver, pancreas, and prostate

In parallel, the first attempts to treat tumours in the liver and pancreas were performed on human patients, under laparotomy or transcutaneously, using long needles.^128,129^

ECT has become the treatment of vertebral metastases that compress the spinal cord, a serious oncological situation quickly leading to paraplegia and inflicting intractable pain on the patient. The expertise at Gustave Roussy involves more than 300 patients today. An initial evaluation of the first 40 patients treated at Gustave Roussy showed, at 1 month, 46% complete regressions and 23% partial responses, knowing that the effect of the ECT session is still progressing in the following two months or more.^130^ An improvement in the quality of life was observed, because the pain, at an average level of 7 on a visual scale of 10 before treatment, was reduced to an average level of 1 in a few days, and in any case less than a month after treatment.

#### Treatment of vascular anomalies

A new application of the same concepts and protocols emerged recently: the treatment of vascular anomalies by bleomycin and reversible electropermeabilization as the method to vectorize the drug.^131^ BEST (Bleomycin ElectroScleroTherapy) is quite efficient in the case of the slow-flow vascular malformations (whether venous, arterial, or lymphatic) and BEET (Bleomycin ElectroEmboTherapy) in the case of the fast-flow arteriovenous malformations. The procedures are disseminating quite rapidly in clinics since they are efficient and safe.

#### History of IRE-based ablation for tumour treatment

IRE was first developed in oncology after studies showed that electric fields exceeding membrane recovery thresholds cause permanent cell death without thermal coagulation.^16,18^ Preclinical work demonstrated sharply demarcated lesions governed by electric-field distribution with preservation of extracellular matrix, vessels, and ducts.^92,93^ These findings established key principles directly relevant to cardiac PFA: tissue response depends on electric-field thresholds rather than temperature, structural scaffolds are preserved, and lesion size is predictable from delivered dose.^91,100^ The same characteristics underpin the myocardial preferentiality and safety profile observed in contemporary PFA procedures.

### Lessons relevant to PFA

#### Understanding tissue-specific electroporation thresholds and safety profiles from non-cardiac experience

The extensive clinical experience accumulated with ECT and, more recently, with BEST, BEET, and gene electrotransfer has generated a large body of data on the delivery of short, high-intensity PEF to human tissues across multiple clinical settings. These studies first established the overall safety of electroporation-based procedures, while also defining the boundaries of that safety, particularly in tissues adjacent to the treatment zone.^132^

In parallel, numerical modelling frameworks originally developed for electrochemotherapy to predict electric field distribution as a function of tissue properties, electrode configuration, and treated volume proved directly transferable to IRE and, subsequently, to cardiac PFA. In particular, the critical role of tissue conductivity heterogeneity in shaping electric field distribution was already being characterized when PFA began to emerge. Adapting this body of knowledge to the cardiac environment nevertheless required additional experimental validation and clinical refinement specific to myocardial anatomy and procedural constraints.^52^

#### Evidence on healing dynamics, inflammation, and long-term tissue remodelling in extracardiac organs

Clinical experience with electrochemotherapy revealed that the effects of PEFs extend beyond transient membrane permeabilization. Local vascular effects and immune responses were identified as important contributors to treatment outcomes. Although immune activation has been observed after both reversible electroporation and IRE, the nature and magnitude of these responses differ, reflecting distinct patterns of cellular injury and cell death.^133^

For cardiac PFA, these observations are relevant because they highlight that electroporation can initiate secondary biological processes that influence tissue response beyond the immediate electrical injury. At the same time, prior oncologic applications provided limited information on healing dynamics and structural remodelling of normal tissues, since ECT is highly selective for tumour cells and spares surrounding healthy tissue at clinically used drug doses. As a result, the translation of electroporation to myocardial ablation required dedicated investigation of myocardial injury, inflammation, and healing, which were not addressed in earlier non-cardiac applications.^132^

## Summary and future directions

PFA represents a paradigm shift in cardiac ablation by exploiting IRE rather than thermal injury to destroy myocardial tissue. Its clinical promise builds on decades of biophysical, molecular, and translational research, combined with system-level innovations tailored to cardiac anatomy and electrophysiology. At the cellular level, short, high-intensity pulses induce TMP, forming nanoscale pores in the lipid bilayer. Membranes may reseal (reversible electroporation) or fail to recover, causing IRE and cell death. The transition is progressive and dose-dependent, explaining the spatial heterogeneity of lesions, with irreversible injury at the core and reversible effects at the periphery.

Tissue architecture and electrical heterogeneity shape the electric field distribution, as differences in conductivity, permittivity, cell orientation, and extracellular composition create regions of enhanced susceptibility or protection. These effects are pronounced in the heart, where fibrosis, scar, infarcted tissue, and blood-filled cavities complicate lesion prediction, making modelling and experimental validation essential. Cardiac PFA builds on lessons from other electroporation-based therapies, including ECT, tumour IRE, and gene transfer, which established safety and dose–response relationships but offered limited insight into normal tissue remodelling.

Looking forward, several open questions remain central to the evolution of PFA. These include optimization of ablation in non-homogeneous myocardium, the role of preheating or combined RF–PFA strategies, refinement of electroporation thresholds in diseased tissue, and continued innovation in waveform design and catheter technology. In parallel, important knowledge gaps persist at the basic science level, particularly regarding cardiomyocyte-specific mechanisms of membrane repair, delayed cell death, and the transition from reversible to irreversible injury. Addressing these challenges will be critical to fully realizing the potential of PFA as a precise, durable, and safe modality for cardiac ablation.

## Data Availability

No new data were generated or analysed in support of this article.
